# Ultrasound findings and clinical testing for preoperative diagnosis of long head of the biceps pathology

**DOI:** 10.1002/jeo2.70430

**Published:** 2025-09-18

**Authors:** Jacques Guery, Maxime Antoni, ReSurg, Chinyelum Agu, Floris van Rooij, Mo Saffarini, David Gallinet

**Affiliations:** ^1^ Polyclinique du Val de Loire ELSAN Nevers France; ^2^ SoFEC ‐ French Shoulder and Elbow Society Paris France; ^3^ Clinique de l'Orangerie, ELSAN Strasbourg France; ^4^ ReSurg SA Nyon Switzerland; ^5^ Clinique Saint Vincent ELSAN Besancon France; ^6^ Centre Epaule Main Besançon Besançon France

**Keywords:** arthroscopy, biceps, diagnosis, rotator cuff, shoulder, ultrasound

## Abstract

**Purpose:**

To determine whether combining ultrasound (US) observations and clinical tests could substantially improve sensitivity for diagnosis of long head of the biceps tendon (LHBT) pathology compared to ultrasound alone and clinical tests alone.

**Methods:**

The authors retrospectively assessed a consecutive series of 284 patients that underwent arthroscopic rotator cuff repair for isolated supraspinatus tears. LHBT pathology was assessed preoperatively using seven US observations ((i) hypervascularisation, (ii) upper gutter signal, (iii) gutter signal, (iv) upper gutter position, (v) gutter position, (vi) upper gutter surface and (vii) gutter surface) and four clinical tests specific to shoulder injuries (Speed, Yergason, Kibler tests and bicipital groove tenderness). Binary outcomes of each assessment were combined to calculate the diagnostic accuracy using intraoperative arthroscopy as reference.

**Results:**

The study cohort comprised 246 patients aged 57.5 ± 8.8 years at index surgery. A total of 56 combinations were tested to obtain the best diagnostic algorithm for detection of LHBT pathologies. Of the 13 combinations with a sensitivity (Se) ≥ 0.85, only four had a specificity (Sp) ≥ 0.25. The ‘tenderness or bicipital gutter surface area’ combination achieved the highest sensitivity (Se, 0.93; Sp, 0.25), followed by the ‘speed or upper bicipital gutter surface area’ combination (Se, 0.87; Sp, 0.28), the ‘tenderness or upper bicipital gutter surface area’ combination (Se, 0.87; Sp, 0.33), and finally the ‘kibler or bicipital gutter surface area’ combination (Se, 0.85; Sp, 0.35).

**Conclusion:**

For the diagnosis of LHBT pathology, using a combination of ultrasound and clinical tests grants higher sensitivity compared to ultrasound or clinical tests alone. The clinical relevance of these findings is that using any combination, 85%–93% of pathologic LHBTs would be correctly diagnosed, while 65%–75% of healthy LHBTs could be misdiagnosed as pathologic.

**Level of Evidence:**

Level IV, diagnostic accuracy study.

**Abbreviations**
FN: false negative
FP: false positive
LHBT: long head of the biceps tendon
MRI: magnetic resonance imaging
NPV: negative predictive value
PPV: positive predictive value
RCR: rotator cuff repair
RCT: rotator cuff tear
Se: sensitivity
Sp: specificity
TN: true negative
TP: true positive

## INTRODUCTION

Pathologies of the long head of the biceps tendon (LHBT) are often concomitant with traumatic and degenerative rotator cuff tears (RCTs), and often result in pain, inflammation or instability [12]. Choosing whether to preserve the LHBT when repairing RCTs, rather than performing either tenodesis or tenotomy is controversial [7, 14]. There is no consensus on whether to perform systematic tenodesis or tenotomy regardless of whether the LHBT is normal or pathologic [1, 11, 21], while others recommend performing tenodesis or tenotomy only in shoulders with LHBT pathology [2, 8, 10, 12, 15, 20, 22], as there are risks and side effects to performing either procedure.

Recent studies have demonstrated that leaving a pathologic LHBT untreated can compromise outcomes of rotator cuff repair (RCR) and lead to patient dissatisfaction. For this reason, preoperative diagnostic tools should aim to maximise sensitivity (reliability at detecting/ruling‐in pathology), even if it compromises specificity (reliability at eliminating/ruling‐out pathology). In a previously published study, assessing the same initial patient cohort, the combination of magnetic resonance imaging (MRI) observations and clinical tests could improve sensitivity for diagnosis of LHBT pathology, compared to MRI tests alone, as it substantially improved the sensitivity (Se, 0.88) but yielded low specificity (Sp, 0.20) [9]. However, ultrasound (US) examination could be faster and cheaper to detect LHBT pathologies, especially when used in combination with clinical tests.

The purpose of the present study was therefore to determine whether combining US observations and clinical tests could substantially improve sensitivity for diagnosis of LHBT pathology [compared to US alone and clinical tests alone]. The combination of specific US observations with clinical tests could enable reliable and user‐friendly diagnosis, and faster shared decision‐making between patient and surgeon regarding LHBT procedures. Therefore, the hypothesis was that a combination of US observations and clinical tests would grant higher sensitivity compared to US observations alone or clinical tests alone.

## METHODS

**Figure 1:**
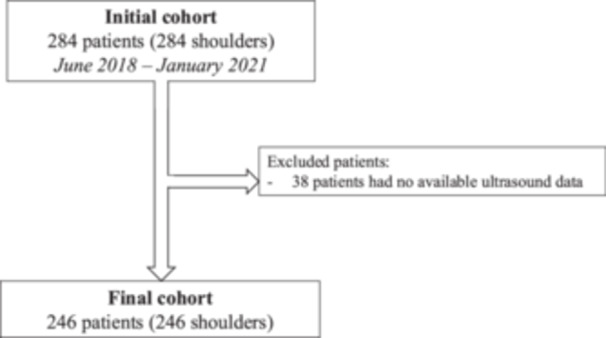
Flowchart of the study cohort.

The authors retrospectively assessed a consecutive series of 284 patients that had arthroscopic RCR of the supraspinatus, by 14 orthopaedic surgeons at 12 centers, between June 2018 and January 2021 (Figure 1). Patients were included if they underwent arthroscopic RCR for isolated supraspinatus tears. Patients were excluded if they did not undergo preoperative ultrasound assessment (*n* = 38). This left a study cohort of 246 patients (246 shoulders) in which biceps status was assessed intraoperatively as either pathologic or healthy during arthroscopy, considered to be the ‘gold‐standard’ diagnosis. All patients provided informed consent, and the study was approved by the local ethics committee (IRB: 2018‐A01382‐53).

### Ultrasound imaging

**Figure 2:**
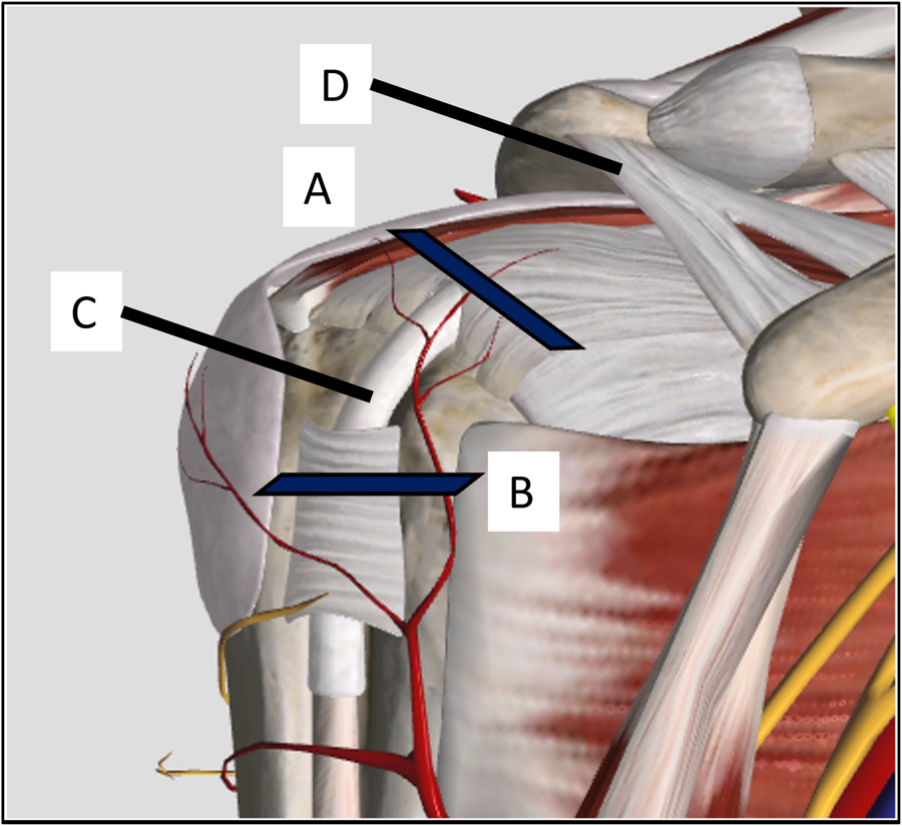
Bicipital gutter positions. A—Upper gutter; B—Gutter; C—long head of the biceps tendon; D—Coracoacromial ligament.

The presence of LHBT pathology was routinely assessed preoperatively on US by each surgeon using seven observations with the following criteria: (i) if positive Doppler vascularisation was found (hypervascularisation), (ii) if the echogenicity was heterogenous (hyper‐ or hypo‐echoic) in the proximal segment of the bicipital gutter (upper gutter signal) (Figure 2), (iii) if the echogenicity was heterogenous (hyper‐ or hypo‐echoic) in the distal segment of the bicipital gutter (gutter signal), (iv) if the LHBT was presented a lateral or medial luxation in the proximal segment of the bicipital gutter (upper gutter position), (v) if the LHBT was presented a lateral or medial luxation in the distal segment of the bicipital gutter (gutter position), (vi) if the LHBT cross sectional surface area was ≥20 mm^2^ in the proximal segment of the bicipital gutter (upper gutter surface) and (vii) if the LHBT cross sectional surface area was ≥13 mm^2^ in the distal segment of the bicipital gutter (gutter surface).

### Clinical assessment

Patients were preoperatively assessed by each surgeon (not blinded to US assessment) using four clinical tests to diagnose shoulder injuries: Speed test [3], Yergason test [4], Kibler test [16] and bicipital groove tenderness. Each test result was presented as either positive in case of pain and therefore suspected pathologic LHBT, or negative if no pain was reported.

### Combining ultrasound observations with clinical tests

The binary outcomes of the seven ultrasound observations and the four clinical tests were combined in various ways, using Boolean operators ‘and/or’ to identify combinations resulting in the best sensitivity using intraoperative arthroscopic findings as the ‘gold‐standard’. The LHBT was either considered pathologic or healthy. The numbers of true positives (TP), true negatives (TN), false positives (FP) and false negatives (FN) were calculated for 56 combinations of US observations and clinical tests to measure sensitivity and specificity (Supplementary Material S2).

### Statistical analysis

No a priori sample size calculation was performed. The sample size was determined based on the number of eligible cases available during the study period. Descriptive statistics were used to summarise the data. The sensitivity (Se), specificity (Sp), accuracy (Acc), positive predictive value (PPV) and negative predictive value (NPV) were calculated for each of the 56 combinations of US observations and clinical tests. Data was presented as means, standard deviations and ranges for descriptive data. Diagnostic accuracy results were presented as TPs, TNs, FPs, FNs, Se, Sp, Acc, PPV, NPV, as well as the sum of Se+Sp. Statistical analyses were performed using R version 4.2.3 (R Foundation for Statistical Computing).

## RESULTS

The study cohort comprised 246 patients (128 men and 118 women) aged 57.5 ± 8.8 years (range: 30–76) at index surgery, with a BMI of 26.6 ± 4.3 (range: 18.0–42.8) (Supplementary Material S1). Of the 246 patients, 168 were operated on their dominant shoulder (68), and 40 were smokers (16). Patients had a preoperative mean Constant score of 54.8 ± 13.1, and subjective shoulder value (SSV) score of 50.6 ± 15.3 (Table 1).

**Table 1:** Preoperative data.

	NA		Final cohort (*n* = 246)		
			Mean ± SD	Range	IQR
			*N* (%)		
Constant score	0	(0%)	54.8 ± 13.1	20–86	17
SSV	6	(2%)	50.6 ± 15.3	10–90	20
Range of motion					
Passive forward elevation	14	(6%)	168 ± 15.5	90–180	20
Passive abduction	18	(7%)	156 ± 23.7	80–180	30
Passive external rotation 1	14	(6%)	55 ± 16.3	0–90	25
Passive external rotation 2	54	(22%)	84 ± 12.8	10–120	10
Active forward elevation	0	(0%)	151 ± 28.2	50–180	30
Active abduction	10	(4%)	138 ± 33.6	45–180	40
Active external rotation 1	1	(0%)	47 ± 17.5	0–90	20
Active external rotation 2	44	(18%)	77 ± 15.9	0–100	20
Active internal rotation	8	(3%)			
(0) Grand trochanter			4 (2%)		
(2) Buttock			23 (10%)		
(4) Sacrum			7 (3%)		
(6) L3			72 (30%)		
(8) T12			92 (39%)		
(10) T7			39 (16%)		
C7			1 (0%)		

Abbreviations: C7, cervical vertabra 7; IQR, interquartile range; L3, lumbar vertabra 3; *N*, cohort size; SD, standard deviation; SSV, subjective shoulder value; T7, thoracic vertabra 7; T12, thoracic vertabra 12*;*

### Single criterion tests

Clinical testing resulted in 171 (70%) positive diagnoses for LHBT pathology using the Speed test, 74 (30%) using the Yergason test, 110 (45%) using the Kibler test and 157 (65%) using the bicipital groove tenderness test. Ultrasound observations found 18 (7%) positive LHBT pathology diagnoses according to hypervascularisation, 119 (49%) using upper gutter signal, 109 (44%) using gutter signal, 243 (100%) using upper gutter position, 21 (9%) using gutter position, 79 (33%) using upper gutter surface and 123 (51%) using gutter surface (Table 2). The speed test (0.75) and the bicipital groove tenderness test had the highest sensitivity (0.75) (Table 3).

**Table 2:** Diagnostic findings.

	Gold standard	Clinical tests				Ultrasound evaluations						
	Arthroscopic findings	Speed	Yergason	Kibler	Tenderness on palpation	Hypervascularisation	Upper gutter signal	Gutter signal	Upper gutter position	Gutter position	Upper gutter surface	Gutter surface
Positive diagnosis for LHBT pathology	109	171	74	110	157	18	119	109	243	21	79	123
Negative diagnosis for LHBT pathology	137	74	169	133	84	226	123	136	0	223	160	116
True positives		82	35	59	79	8	55	49	107	15	45	64
True negatives		47	96	85	57	125	70	76	0	130	99	74
False positives		89	39	51	78	10	64	60	136	6	34	59
False negatives		27	73	48	27	101	53	60	0	93	61	42

Abbreviation: LHBT, long head of the biceps tendon.

**Table 3:** Diagnostic findings.

						Sensitivity		Specificity		Accuracy		PPV		NPV		Se+Sp
	*N*	TP	FP	FN	TN	Est		Est.		Est.		Est.		Est.		
Clinical test																
Speed	245	82	89	27	47	0.75	(0.66–0.83)	0.35	(0.27–0.43)	0.53	(0.46–0.59)	0.48	(0.40–0.56)	0.64	(0.52–0.74)	1.10
Yergason	243	35	39	73	96	0.32	(0.24–0.42)	0.71	(0.63–0.79)	0.54	(0.47–0.60)	0.47	(0.36–0.59)	0.57	(0.49–0.64)	1.04
Kibler	243	59	51	48	85	0.55	(0.45–0.65)	0.63	(0.54–0.71)	0.59	(0.53–0.65)	0.54	(0.44–0.63)	0.64	(0.55–0.72)	1.18
Tenderness	241	79	78	27	57	0.75	(0.65–0.82)	0.42	(0.34–0.51)	0.56	(0.50–0.63)	0.50	(0.42–0.58)	0.68	(0.57–0.78)	1.17
Ultrasound evaluation																
Hypervascularisation	244	8	10	101	125	0.07	(0.03–0.14)	0.93	(0.87–0.96)	0.55	(0.48–0.61)	0.44	(0.22–0.69)	0.55	(0.49–0.62)	1.00
Upper gutter signal	242	55	64	53	70	0.51	(0.41–0.61)	0.52	(0.43–0.61)	0.52	(0.45–0.58)	0.46	(0.37–0.56)	0.57	(0.48–0.66)	1.03
Gutter signal	245	49	60	60	76	0.45	(0.35–0.55)	0.56	(0.47–0.64)	0.51	(0.45–0.57)	0.45	(0.35–0.55)	0.56	(0.47–0.64)	1.01
Upper gutter position	243	107	136	0	0	1.00	(0.97–1.00)	0.00	(0.00–0.03)	0.44	(0.38–0.51)	0.44	(0.38–0.51)	NaN	(0.00–1.00)	1.00
Gutter position	244	15	6	93	130	0.14	(0.08–0.22)	0.96	(0.91–0.98)	0.59	(0.53–0.66)	0.71	(0.48–0.89)	0.58	(0.52–0.65)	1.09
Upper gutter surfaces	239	45	34	61	99	0.42	(0.33–0.52)	0.74	(0.66–0.82)	0.60	(0.54–0.67)	0.57	(0.45–0.68)	0.62	(0.54–0.69)	1.17
Gutter surfaces	239	64	59	42	74	0.60	(0.50–0.70)	0.56	(0.47–0.64)	0.58	(0.51–0.64)	0.52	(0.43–0.61)	0.64	(0.54–0.73)	1.16

Abbreviations: Est., estimation; FN, false negative; FP, false positive; N, cohort size; NPV, negative predictive value; PPV, positive predictive value; Se, sensitivity; Sp, specificity; TN, true negative; TP, true positive.

**Figure 3:**
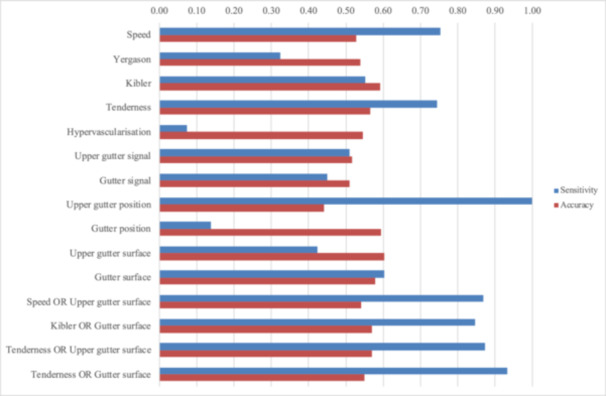
Bar charts of the sensitivity and accuracy of ultrasound observations, clinical tests and a combination thereof.

For ultrasound, the upper gutter position had the highest sensitivity but a specificity of 0% (Se, 1.00; Sp, 0.00), while gutter surface (Se, 0.60; Sp, 0.56) and upper gutter signal (Se, 0.51; Sp, 0.52) had moderate sensitivity and specificity (Table 3, Figure 3).

### Multiple criteria test

A total of 56 combinations were tested to obtain the best diagnostic algorithm for detection of LHBT pathologies. A total of 13 combinations reached a sensitivity ≥0.85, but had a specificity ≤0.35 (Table 4). The ‘speed or upper gutter position’, ‘yergason or upper gutter position’, ‘kibler or upper gutter position’ and ‘tenderness or upper gutter position’ all had the highest sensitivity but the lowest specificity (Se, 1.00; Sp, 0.00).

**Table 4:** Diagnostic findings for two criteria combinations (sensitivity > 0.70).

	*N*	TP	FP	FN	TN	Sensitivity		Specificity		Accuracy		PPV		NPV		Se+Sp
						Est.		Est.		Est.		Est.		Est.		
Speed OR hypervascularisation	244	84	89	25	46	0.77	(0.68–0.85)	0.34	(0.26–0.43)	0.53	(0.47–0.60)	0.49	(0.41–0.56)	0.65	(0.53–0.76)	1.11
Speed OR upper gutter signal	242	94	109	14	25	0.87	(0.79–0.93)	0.19	(0.12–0.26)	0.49	(0.43–0.56)	0.46	(0.39–0.53)	0.46	(0.47–0.79)	1.06
Speed OR gutter signal	245	93	108	16	28	0.85	(0.77–0.91)	0.21	(0.14–0.28)	0.49	(0.43–0.56)	0.46	(0.39–0.53)	0.64	(0.48–0.78)	1.06
Speed AND upper gutter position	243	81	89	26	47	0.76	(0.66–0.83)	0.35	(0.27–0.43)	0.53	(0.46–0.59)	0.48	(0.40–0.55)	0.64	(0.52–0.75)	1.10
**Speed OR upper gutter position**	243	107	136	0	0	1.00	(0.97–1.00)	0.00	(0.00–0.03)	0.44	(0.38–0.51)	0.44	(0.38–0.51)	NaN	(0.00–1.00)	1.00
Speed OR gutter position	244	84	92	24	44	0.78	(0.69–0.85)	0.32	(0.25–0.41)	0.52	(0.46–0.59)	0.48	(0.40–0.55)	0.65	(0.52–0.76)	1.10
Speed OR upper gutter surface	239	92	96	14	37	0.87	(0.79–0.93)	0.28	(0.20–0.36)	0.54	(0.47–0.60)	0.49	(0.42–0.56)	0.73	(0.58–0.84)	1.15
Speed AND gutter surface	239	81	88	25	45	0.76	(0.67–0.84)	0.34	(0.26–0.43)	0.53	(0.46–0.59)	0.48	(0.40–0.56)	0.64	(0.52–0.75)	1.10
Speed OR gutter surface	239	98	105	8	28	0.92	(0.86–0.97)	0.21	(0.14–0.29)	0.53	(0.46–0.59)	0.48	(0.41–0.55)	0.78	(0.61–0.90)	1.14
Yergason OR upper gutter position	241	106	135	0	0	1.00	(0.97–1.00)	0.00	(0.00–0.03)	0.44	(0.38–0.50)	0.44	(0.38–0.50)	NaN	(0.00–1.00)	1.00
Yergason OR gutter surface	237	80	83	25	49	0.76	(0.67–0.84)	0.37	(0.29–0.46)	0.54	(0.48–0.61)	0.49	(0.41–0.57)	0.66	(0.54–0.77)	1.13
Kibler OR upper gutter signal	240	78	91	28	43	0.74	(0.64–0.82)	0.32	(0.24–0.41)	0.50	(0.44–0.57)	0.46	(0.38–0.54)	0.61	(0.48–0.72)	1.06
**Kibler OR gutter signal**	243	78	90	29	46	0.73	(0.63–0.81)	0.34	(0.26–0.42)	0.51	(0.45–0.57)	0.46	(0.39–0.54)	0.61	(0.49–0.72)	1.07
Kibler OR upper gutter position	241	105	136	0	0	1.00	(0.97–1.00)	0.00	(0.00–0.03)	0.44	(0.37–0.50)	0.44	(0.37–0.50)	NaN	(0.00–1.00)	1.00
Kibler OR upper gutter surface	237	80	69	24	64	0.77	(0.68–0.85)	0.48	(0.39–0.57)	0.61	(0.54–0.67)	0.54	(0.45–0.62)	0.73	(0.62–0.82)	1.25
Kibler OR gutter surface	237	88	86	16	47	0.85	(0.76–0.91)	0.35	(0.27–0.44)	0.57	(0.50–0.63)	0.51	(0.43–0.58)	0.75	(0.62–0.85)	1.20
Tenderness OR hypervascularisation	240	82	80	24	54	0.77	(0.68–0.85)	0.40	(0.32–0.49)	0.57	(0.50–0.63)	0.51	(0.43–0.59)	0.69	(0.58–0.79)	1.18
Tenderness OR upper gutter signal	238	91	103	14	30	0.87	(0.79–0.93)	0.23	(0.16–0.31)	0.51	(0.44–0.57)	0.47	(0.40–0.54)	0.68	(0.52–0.81)	1.09
Tenderness OR gutter signal	241	92	102	14	33	0.87	(0.79–0.93)	0.24	(0.17–0.33)	0.52	(0.45–0.58)	0.47	(0.40–0.55)	0.70	(0.55–0.83)	1.11
Tenderness AND upper gutter position	239	78	78	26	57	0.75	(0.66–0.83)	0.42	(0.34–0.51)	0.56	(0.50–0.63)	0.50	(0.42–0.58)	0.69	(0.58–0.78)	1.17
Tenderness OR upper gutter position	239	104	135	0	0	1.00	(0.97–1.00)	0.00	(0.00–0.03)	0.44	(0.37–0.50)	0.44	(0.44–0.50)	NaN	(0.00–1.00)	1.00
Tenderness OR gutter position	240	81	81	24	54	0.77	(0.68–0.85)	0.40	(0.32–0.49)	0.56	(0.50–0.63)	0.50	(0.42–0.58)	0.69	(0.58–0.79)	1.17
**Tenderness OR upper gutter surface**	235	90	88	13	44	0.87	(0.79–0.93)	0.33	(0.25–0.42)	0.57	(0.50–0.63)	0.51	(0.43–0.58)	0.77	(0.64–0.87)	1.21
Tenderness AND gutter surface	235	77	77	26	55	0.75	(0.65–0.83)	0.42	(0.33–0.51)	0.56	(0.50–0.63)	0.50	(0.42–0.58)	0.68	(0.57–0.78)	1.16
**Tenderness OR gutter surface**	235	96	99	7	33	0.93	(0.86–0.97)	0.25	(0.18–0.33)	0.55	(0.48–0.61)	0.49	(0.42–0.56)	0.83	(0.67–0.93)	1.18

*Note*: Values in bold represent the combinations considered for final selection. Abbreviations: Est., estimation; FN, false negative; FP, false positive; *N*, cohort size; NPV, negative predictive value; PPV, positive predictive value; Se, sensitivity; Sp, specificity; TN, true negative; TP, true positive.

Of the 13 combinations with a sensitivity ≥0.85, only four had a specificity ≥0.25. The ‘tenderness or gutter surface’ combination achieved the highest sensitivity (Se, 0.93; Sp, 0.25), followed by the ‘speed or upper gutter surface’ combination (Se, 0.87; Sp, 0.28), the ‘tenderness or upper gutter surface’ combination (Se, 0.87; Sp, 0.33), and finally the ‘kibler or gutter surface’ combination (Se, 0.85; Sp, 0.35).

Of these combinations, the best diagnostic accuracies were achieved for the ‘tenderness or upper gutter surface’ combination (Acc, 0.57), and by the ‘kibler or gutter surface’ combination (Acc, 0.57), and finally the ‘tenderness or gutter surface’ combination (Acc, 0.55).

## DISCUSSION

The most important findings of this study were that, for the diagnosis of LHBT pathology using ultrasound and clinical tests, with a sensitivity ≥0.85 and a specificity ≥0.25, the best diagnostic accuracies were achieved for the ‘tenderness or upper gutter surface’ combination (Acc, 0.57), followed by the ‘kibler or gutter surface’ combination (Acc, 0.57), and finally the ‘tenderness or gutter surface’ combination (Acc, 0.55). These findings confirm the hypothesis that a combination of US observations and clinical tests would grant higher sensitivity compared to US observations alone or clinical tests alone. The clinical relevance of these findings is that using any combination, 85%–93% of pathologic LHBTs would be correctly diagnosed, while 65%–75% of healthy LHBTs could be misdiagnosed as pathologic. Therefore, clinicians that use US for diagnosis of LHBT pathology should focus on the gutter or upper gutter surface, and ideally inspect for tenderness or perform the Kibler test.

Nowadays, there are a number of clinical tests available for the assessment of RCT, and more specifically of LHBT pathology. In a systematic review from 2017 by Rosas et al. [19], the authors investigated the diagnostic accuracy of clinical test used for detection of LHBT pathology and found the highest sensitivity for the bear hug test (Se, 0.79), followed by the uppercut test (Se, 0.73). The tenderness on palpation (Se, 0.57), speed test (Se, 0.54) and yergason test (Se, 0.41) only came in 4th, 5th and 6th place respectively, of the seven tests available in the review. A more recent meta‐analysis by Courage et al. [5] reported that the Speed test had a sensitivity ranging from 0.32 to 0.61, and the Yergason test had sensitivity ranging from 0.37 to 0.43 for diagnosis of LHBT pathology. The diagnostic values reported in the studies are insufficient for reliable preoperative diagnosis of LHBT pathology. In comparison, the present study found sensitivities ranging from 0.32 to 0.75 for individual clinical tests indicating that clinical tests alone do not provide adequate sensitivity for the assessment of LHBT pathology.

Imaging modalities for the diagnosis of LHBT pathologies have also been investigated in the literature. In the meta‐analysis by Courage et al. [5], the authors found that US had uncertain sensitivity (range: 0.27–1.00), with a pooled sensitivity of 0.70. Moreover, recent systematic reviews of Courage et al. [5] and Lalevée et al. [18] revealed minimal differences in diagnostic accuracy of US versus MRI for partial tears (US, 0.27–0.71; MRI, 0.20–0.82). Furthermore, a meta‐analysis by Gryftopoulos et al. [13] compared MRI to US in terms of diagnostic accuracy for the detection of RCTs, and found that US demonstrated greater sensitivity (MRI, 85.9%; US, 89.7) and specificity (MRI, 89.1%; US, 91.0) than MRI. A study by Gallinet et al. [9] which aimed to determine whether MRI observations and clinical tests could improve sensitivity for diagnosis of LHBT pathology, found a benefit in combining the testing modalities. The MRI observations had sensitivities ranging from 0.12 to 0.68. The present study aims to combine clinical test to several US observations with sensitivities ranging from 0.07 to 0.60, with the exception of the ‘upper gutter position’ which yielded a sensitivity of 1.00, but a specificity of 0.00. Furthermore, US observations such as hypervascularisation and gutter position had very low sensitivity and therefore have minimal correlation with arthroscopic findings on a case‐by‐case basis, however, when combining these with clinical tests, the sensitivity greatly improves. Obtaining better sensitivity when combining clinical tests to US rather than MRI would allow considerably faster and cheaper examinations to perform, and therefore could facilitate decision‐making during initial patient assessment.

While the speed and tenderness tests by themselves had sensitivities of 0.75, the addition of US evaluations increased the sensitivity in 9 of the 14 combinations for both tests. The findings of the present study, however, show that an increase in sensitivity leads to a decrease in specificity when combining clinical tests and US observations. Innovative diagnostic methodologies rely increasingly on combinations of findings using different methods or modalities to reduce the margin of errors. The present study is the first to investigate a combination of clinical tests and ultrasound findings for diagnosis of LHBT pathology. Accurate preoperative detection of LHBT pathology is important, as it allows to manage patients' expectations and intraoperative time. Nevertheless, intraoperative arthroscopic findings should remain the final assessment of the intra‐articular portion of LHBT, during which any diagnostic error could be correctly rectified and treated.

When combining clinical tests to MRI observations, Gallinet et al. [9] found that a combination of clinical tests and MRI findings (Se, 0.88) resulted in a higher sensitivity than clinical tests (Se, 0.74) or MRI findings alone (Se, 0.68). Furthermore, in a systematic review by Rosas et al. [19], the authors developed a practical, evidence‐based algorithm to accurately diagnose patients with LHBT pathology using US observations and clinical evaluations, and identified the parallel combination (or) of the uppercut test and US to have the highest sensitivity (Se, 0.97), followed by the parallel combinations of tenderness on palpation and US (Se, 0.95), and Speed and US (Se, 0,94). However, Rosas et al. [19] only used a single US observation to examine the LHBT. In comparison, the present study identified the best combinations as ‘tenderness (on palpation) or gutter surface’ (Se, 0.93), followed by ‘speed or upper gutter surface’ (Se, 0.87), ‘tenderness or upper gutter surface’ (Se, 0.87), and finally ‘kibler or gutter’ (Se, 0.85). These findings revealed that combinations of clinical tests and US findings resulted in a higher sensitivity than clinical tests alone (Se, 0.32–0.75) or US findings alone (Se, 0.07–0.60), with the exception of the ‘upper gutter position’ (Se, 1.00; Sp, 0.00) due to giving only positive results, even in cases of a healthy LHBT (n, 243; TP, 107; TN, 0; FP, 136; FN, 0).

The findings of the present study should be interpreted with the following limitation in mind. First, as some ultrasound criteria can be subjective to interpret, it is possible that US readings might have differed between the 14 surgeons since interobserver repeatability was not assessed, the ultimate diagnostic repeatability of the combinations cannot be ascertained, although the authors had sufficient confidence in the repeatability of ultrasound [6, 17]. Second, the clinical tests used can show great variability in sensitivity and specificity, as patient symptoms such as pain, and clinician experience can influence the outcomes of the examinations. Third, all of the LHBT pathologies were grouped together, without differentiating between tendinitis, instability, partial tear, complete tear, or SLAP lesions, which may limit the clinical relevance. Fourth, it is possible that patient characteristics (age, BMI, shoulder dominance) have a confounding impact on diagnostic accuracy, and therefore future studies should investigate these associations. Finally, the present study did not include a control group as all patients were retrospectively included after undergoing RCR for symptomatic supraspinatus tears. However, inclusion of asymptomatic patients without supraspinatus tears would not permit calculations of diagnostic accuracy as these patients would not have undergone arthroscopic evaluation. The strength of the present study is that it is the first to investigate the combination of clinical tests and ultrasound observations for the diagnosis of LHBT pathology, which could provide greater diagnostic value for clinicians. Furthermore, these findings can help to limit unnecessary tenodesis or tenotomy procedures in nonpathologic LHBTs, and thus improve patient quality of life by preserving them from undesirable effects such as postoperative pain, or the Popeye sign, and preventing unnecessary surgical time extension.

## CONCLUSION

For the diagnosis of LHBT pathology, using a combination of ultrasound and clinical tests grants higher sensitivity compared to ultrasound or clinical tests alone. The clinical relevance of these findings is that using any combination, 85%–93% of pathologic LHBTs would be correctly diagnosed, while 65%–75% of healthy LHBTs could be misdiagnosed as pathologic.

## AUTHOR CONTRIBUTIONS

**David Gallinet, Jacques Guery**: Funding acquisition; data collection; methodology; supervision; validation. **Maxime Antoni**: Data collection; methodology; validation. **Chinyelum Agu, Floris van Rooij, Mo Saffarini**: Methodology; manuscript writing; formal analysis; validation.

## COLLABORATORS OF RESURG

Chinyelum Agu, Floris van Rooij and Mo Saffarini.

## CONFLICT OF INTEREST STATEMENT

David Gallinet reports consulting and royalties from moveUP outside the submitted work. Maxime Antoni reports fees from ConMed and fees and royalties FX Shoulder Solutions outside the submitted work. Jacques Guery reports fees from moveUP outside of the submitted work. The remaining authors declare no conflicts of interest.

## ETHICS STATEMENT

All patients provided informed consent and the study was approved by the local ethics committee (IRB: 2018‐A01382‐53).

## Supporting information


**Supplementary Material 1**: Patient demographics. **Supplementary Material 2**: Diagnostic findings for 2 criteria combinations.

## Data Availability

All data are available upon reasonable request.
