# Supplementary material for: Ultrasound findings and clinical testing for preoperative diagnosis of long head of the biceps pathology
Source: J Exp Orthop. 2025 Sep 18;12(3):e70430. doi: 10.1002/jeo2.70430 (PMC12445285; doi:10.1002/jeo2.70430)
Supplement: Supplementary file 1 — Supplementary Material 1: Patient demographics. Supplementary Material 2: Diagnostic findings for 2 criteria combinations. [file JEO2-12-e70430-s001.docx]

**Supplementary material 1:**

| **Patient demographics** | | | | | | |  | | |  | | |  | | |  | | |  | | |  | | |  | | |  | | |  | | |  | | |  | |  | | | | |  | | | |  | | | | | |  |
| --- | --- | --- | --- | --- | --- | --- | --- | --- | --- | --- | --- | --- | --- | --- | --- | --- | --- | --- | --- | --- | --- | --- | --- | --- | --- | --- | --- | --- | --- | --- | --- | --- | --- | --- | --- | --- | --- | --- | --- | --- | --- | --- | --- | --- | --- | --- | --- | --- | --- | --- | --- | --- | --- | --- |
|  |  |  |  | |  | | |  | | |  | | |  | | |  | | |  | | |  | | |  | | |  | | | |  | |  | |  | | |  | | | | | |  | | |  | | | | | |
|  |  |  |  | |  | | |  | | |  | | |  | | |  | | |  | | |  | | |  | | |  | | | |  | |  | |  | | |  | | | | | |  | | |  | | | | | |
|  |  | **Initial Cohort (n=284)** | | | | | | | | | | | | | | | | | |  | | | **Final Cohort (n=246)** | | | | | | | | | | | | | | | | | | | |  | | | |  | | | | | |  |  |
|  |  | Mean | ± | SD | |  | | | Range | | | | | | | | |  | | | Mean | | | ± | | | SD | | |  | | Range | | | | | | | | | |  | | |  | | | | | |  |  |  |  |
|  |  | N |  | (%) | |  | | |  |  |  |  |  |  |  |  |  |  | | | N | | |  | | | (%) | | |  | |  |  |  |  |  |  |  |  |  |  |  | | |  | | | | | |  |  |  |  |
|  |  |  |  |  | |  | | |  | | |  | | |  | | |  | | |  | | |  | | |  | | |  | |  | | | |  | |  | | |  | | |  | | | | | |  |  |  |  |  |
|  |  |  |  |  | |  | | |  | | |  | | |  | | |  | | |  | | |  | | |  | | |  | |  | | | |  | |  | | |  | | |  | | | | | |  |  |  |  |  |
|  | **Age** | 57.5 | ± | 8.7 | |  | | | 30.1 | | | – | | | 75.9 | | |  | | | 57.5 | | | ± | | | 8.8 | | |  | | 30.1 | | | | – | | 75.9 | | |  | | |  | | | | | |  |  |  |  |  |
|  | **BMI** | 26.7 | ± | 4.3 | |  | | | 17.7 | | | – | | | 42.8 | | |  | | | 26.6 | | | ± | | | 4.3 | | |  | | 18.0 | | | | – | | 42.8 | | |  | | |  | | | | | |  |  |  |  |  |
|  | **Sex** |  |  |  | |  | | |  | | |  | | |  | | |  | | |  | | |  | | |  | | |  | |  | | | |  | |  | | |  | | |  | | | | | |  |  |  |  |  |
|  | Male | 134 |  | (47%) | |  | | |  | | |  | | |  | | |  | | | 128 | | |  | | | (52%) | | |  | |  | | | |  | |  | | |  | | |  | | | | | |  |  |  |  |  |
|  | Female | 150 |  | (53%) | |  | | |  | | |  | | |  | | |  | | | 118 | | |  | | | (48%) | | |  | |  | | | |  | |  | | |  | | |  | | | | | |  |  |  |  |  |
|  | **Dominant shoulder** | 197 |  | (69%) | |  | | |  | | |  | | |  | | |  | | | 168 | | |  | | | (68%) | | |  | |  | | | |  | |  | | |  | | |  | | | | | |  |  |  |  |  |
|  | **Smoking** | 47 |  | (17%) | |  | | |  | | |  | | |  | | |  | | | 40 | | |  | | | (16%) | | |  | |  | | | |  | |  | | |  | | |  | | | | | |  |  |  |  |  |
|  |  |  |  |  | |  | | |  | | |  | | |  | | |  | | |  | | |  | | |  | | |  | |  | | | |  | |  | | |  | | |  | | | | | |  |  |  |  |  |
| *Abbreviations: BMI, Body Mass Index; N, Cohort Size; SD, Standard Deviation;* | | | | | | | | | | | | | | | | | | | | | | | | | | | | | | | | | | | | | | | | | | | | | | | | | | | |  |  |  |

**Supplementary material 2:**

| **Table 4: Diagnostic findings for 2 criteria combinations** | | | | | | | | | | | | | | | | | | | | | | | | | | | | | | | | | | | | | | | | | | | | | | | | | | |  | | | | | | | | |  | | | | | |  | | | | |  | | | | |  | | | | | | |  | | | | | |  | | | | |  | | | |  | | | | | | |  | | | | | | |  | | | |  | | | | | | |  | | | | |  | | | | | |  | | | | | |  | | | | | | |  | | | | | | | |  |  |  |  |  |  |  |
| --- | --- | --- | --- | --- | --- | --- | --- | --- | --- | --- | --- | --- | --- | --- | --- | --- | --- | --- | --- | --- | --- | --- | --- | --- | --- | --- | --- | --- | --- | --- | --- | --- | --- | --- | --- | --- | --- | --- | --- | --- | --- | --- | --- | --- | --- | --- | --- | --- | --- | --- | --- | --- | --- | --- | --- | --- | --- | --- | --- | --- | --- | --- | --- | --- | --- | --- | --- | --- | --- | --- | --- | --- | --- | --- | --- | --- | --- | --- | --- | --- | --- | --- | --- | --- | --- | --- | --- | --- | --- | --- | --- | --- | --- | --- | --- | --- | --- | --- | --- | --- | --- | --- | --- | --- | --- | --- | --- | --- | --- | --- | --- | --- | --- | --- | --- | --- | --- | --- | --- | --- | --- | --- | --- | --- | --- | --- | --- | --- | --- | --- | --- | --- | --- | --- | --- | --- | --- | --- | --- | --- | --- | --- | --- | --- | --- | --- | --- | --- | --- | --- | --- | --- | --- | --- | --- | --- | --- | --- | --- | --- | --- |
|  | | |  | | | | | | | | | | | | | | | | | | | | | | | | | | | | |  | | | | | | |  | |  | | | | |  | | | | | |  | | | | | | | | |  | | | | | |  | | | | |  | | | |  | | | | | | |  | | | | | |  | | | | |  | | | |  | | | | | | |  | | | | | | |  | | | |  | | | | | | |  | | | | |  | | | | | |  | | | | |  | | | | | | |  | | | | | | | | | | | | | | |  |
|  | | |  | | | | | | | | | | | | | | | | | | | | | | | | | | | | |  | | | | | | |  | |  | | | | |  | | | | | |  | | | | | | | | |  | | | | | |  | | | | |  | | | |  | | | | | | |  | | | | | |  | | | | |  | | | |  | | | | | | |  | | | | | | |  | | | |  | | | | | | |  | | | | |  | | | | | |  | | | | |  | | | | | | |  | | | | | | | |  |  |  |  |  |  |  |  |
|  |  | | | | | | N | | | |  | | | | TP | | | | FP | | | FN | | | | TN | | | |  | | | | | Sensitivity | | | | | | | | | | | | | | | | | | | | |  | | | | | | | Specificity | | | | | | | | | | | | | | | | |  | | | | | | Accuracy | | | | | | | | | | | | | | | | |  | | | PPV | | | | | | | | | | | | | | |  | | | | | | NPV | | | | | | | | | | | | | | | | |  | | | | | | | Se+Sp | | | | | | | | | | |
|  |  | | | | | | | |  | | |  | | | |  | | | | |  | | | |  | | | |  | | | |  | | | | Est. | | | | | | | |  | | | | |  | | | | | | | Est. | | | | | | | |  | | | | | | | | | | | | | | | |  | | | Est. | | | | | | | |  | | | | | | |  | | | | | | | | | Est. | | |  | | | | | | | |  | | | | | | | Est. | | | | | | |  | | | | |  | | | | | | |  | | | | | | |  | | | | | |  |  |  |  |
|  | | | |  | | | | | | | | | | | | | | | | | | | | | | | | | | | | | |  | | | | | |  | | |  | | | |  | | | | | | |  | | | | | | | |  | | | | | |  | | | | |  | | | | |  | | | | | | |  | | | | |  | | | | |  | | | | |  | | | | | | |  | | | | | |  | | | |  | | | | | | |  | | | | | |  | | | | |  | | | | | |  | | | | | | |  | | | | | | | | | | | | | |
|  | | | |  | | | | | | | | | | | | | | | | | | | | | | | | | | | | | |  | | | | | |  | | |  | | | |  | | | | | | |  | | | | | | | |  | | | | | |  | | | | |  | | | | |  | | | | | | |  | | | | |  | | | | |  | | | | |  | | | | | | |  | | | | | |  | | | |  | | | | | | |  | | | | | |  | | | | |  | | | | | |  | | | | | | |  | | | | | | | | | | | | | |
|  | | **Speed AND Hypervascularisation** | | | | | | 244 | | | | |  | | | | 6 | | | 9 | | | | 103 | | | 126 | | | |  | | | | | 0.06 | | | | | | | | (0.02 | | | | | – | | | | 0.12) | | | | | |  | | | | | 0.93 | | | | | | (0.88 | | | | | – | | 0.97) | | | | |  | | | | | | 0.54 | | | | | (0.48 | | | | – | | | | 0.60) | | |  | | | | | | 0.40 | | | | (0.16 | | | | | | – | | 0.68) | | | | | | |  | | | 0.55 | | | | | (0.48 | | | | | – | | | | | | | 0.62) | | | |  | | | | 0.99 | | |  | |
|  | | **Speed OR Hypervascularisation** | | | | | | 244 | | | | |  | | | | 84 | | | 89 | | | | 25 | | | 46 | | | |  | | | | | 0.77 | | | | | | | | (0.68 | | | | | – | | | | 0.85) | | | | | |  | | | | | 0.34 | | | | | | (0.26 | | | | | – | | 0.43) | | | | |  | | | | | | 0.53 | | | | | (0.47 | | | | – | | | | 0.60) | | |  | | | | | | 0.49 | | | | (0.41 | | | | | | – | | 0.56) | | | | | | |  | | | 0.65 | | | | | (0.53 | | | | | – | | | | | | | 0.76) | | | |  | | | | 1.11 | | |  | |
|  | | **Speed AND Upper gutter signal** | | | | | | 242 | | | | |  | | | | 43 | | | 43 | | | | 65 | | | 91 | | | |  | | | | | 0.40 | | | | | | | | (0.31 | | | | | – | | | | 0.50) | | | | | |  | | | | | 0.68 | | | | | | (0.59 | | | | | – | | 0.76) | | | | |  | | | | | | 0.55 | | | | | (0.49 | | | | – | | | | 0.62) | | |  | | | | | | 0.50 | | | | (0.39 | | | | | | – | | 0.61) | | | | | | |  | | | 0.58 | | | | | (0.50 | | | | | – | | | | | | | 0.66) | | | |  | | | | 1.08 | | |  | |
|  | | **Speed OR Upper gutter signal** | | | | | | 242 | | | | |  | | | | 94 | | | 109 | | | | 14 | | | 25 | | | |  | | | | | 0.87 | | | | | | | | (0.79 | | | | | – | | | | 0.93) | | | | | |  | | | | | 0.19 | | | | | | (0.12 | | | | | – | | 0.26) | | | | |  | | | | | | 0.49 | | | | | (0.43 | | | | – | | | | 0.56) | | |  | | | | | | 0.46 | | | | (0.39 | | | | | | – | | 0.53) | | | | | | |  | | | 0.46 | | | | | (0.47 | | | | | – | | | | | | | 0.79) | | | |  | | | | 1.06 | | |  | |
|  | | **Speed AND Gutter signal** | | | | | | 245 | | | | |  | | | | 38 | | | 41 | | | | 71 | | | 95 | | | |  | | | | | 0.35 | | | | | | | | (0.26 | | | | | – | | | | 0.45) | | | | | |  | | | | | 0.70 | | | | | | (0.61 | | | | | – | | 0.77) | | | | |  | | | | | | 0.54 | | | | | (0.48 | | | | – | | | | 0.61) | | |  | | | | | | 0.48 | | | | (0.37 | | | | | | – | | 0.60) | | | | | | |  | | | 0.57 | | | | | (0.49 | | | | | – | | | | | | | 0.65) | | | |  | | | | 1.05 | | |  | |
|  | | **Speed OR Gutter signal** | | | | | | 245 | | | | |  | | | | 93 | | | 108 | | | | 16 | | | 28 | | | |  | | | | | 0.85 | | | | | | | | (0.77 | | | | | – | | | | 0.91) | | | | | |  | | | | | 0.21 | | | | | | (0.14 | | | | | – | | 0.28) | | | | |  | | | | | | 0.49 | | | | | (0.43 | | | | – | | | | 0.56) | | |  | | | | | | 0.46 | | | | (0.39 | | | | | | – | | 0.53) | | | | | | |  | | | 0.64 | | | | | (0.48 | | | | | – | | | | | | | 0.78) | | | |  | | | | 1.06 | | |  | |
|  | | **Speed AND Upper gutter position** | | | | | | 243 | | | | |  | | | | 81 | | | 89 | | | | 26 | | | 47 | | | |  | | | | | 0.76 | | | | | | | | (0.66 | | | | | – | | | | 0.83) | | | | | |  | | | | | 0.35 | | | | | | (0.27 | | | | | – | | 0.43) | | | | |  | | | | | | 0.53 | | | | | (0.46 | | | | – | | | | 0.59) | | |  | | | | | | 0.48 | | | | (0.40 | | | | | | – | | 0.55) | | | | | | |  | | | 0.64 | | | | | (0.52 | | | | | – | | | | | | | 0.75) | | | |  | | | | 1.10 | | |  | |
|  | | **Speed OR Upper gutter position** | | | | | | 243 | | | | |  | | | | 107 | | | 136 | | | | 0 | | | 0 | | | |  | | | | | 1.00 | | | | | | | | (0.97 | | | | | – | | | | 1.00) | | | | | |  | | | | | 0.00 | | | | | | (0.00 | | | | | – | | 0.03) | | | | |  | | | | | | 0.44 | | | | | (0.38 | | | | – | | | | 0.51) | | |  | | | | | | 0.44 | | | | (0.38 | | | | | | – | | 0.51) | | | | | | |  | | | NaN | | | | | (0.00 | | | | | – | | | | | | | 1.00) | | | |  | | | | 1.00 | | |  | |
|  | | **Speed AND Gutter position** | | | | | | 244 | | | | |  | | | | 12 | | | 3 | | | | 96 | | | 133 | | | |  | | | | | 0.11 | | | | | | | | (0.06 | | | | | – | | | | 0.19) | | | | | |  | | | | | 0.98 | | | | | | (0.94 | | | | | – | | 1.00) | | | | |  | | | | | | 0.59 | | | | | (0.53 | | | | – | | | | 0.66) | | |  | | | | | | 0.80 | | | | (0.52 | | | | | | – | | 0.96) | | | | | | |  | | | 0.58 | | | | | (0.51 | | | | | – | | | | | | | 0.65) | | | |  | | | | 1.09 | | |  | |
|  | | **Speed OR Gutter position** | | | | | | 244 | | | | |  | | | | 84 | | | 92 | | | | 24 | | | 44 | | | |  | | | | | 0.78 | | | | | | | | (0.69 | | | | | – | | | | 0.85) | | | | | |  | | | | | 0.32 | | | | | | (0.25 | | | | | – | | 0.41) | | | | |  | | | | | | 0.52 | | | | | (0.46 | | | | – | | | | 0.59) | | |  | | | | | | 0.48 | | | | (0.40 | | | | | | – | | 0.55) | | | | | | |  | | | 0.65 | | | | | (0.52 | | | | | – | | | | | | | 0.76) | | | |  | | | | 1.10 | | |  | |
|  | | **Speed AND Upper gutter surface** | | | | | | 239 | | | | |  | | | | 34 | | | 26 | | | | 72 | | | 107 | | | |  | | | | | 0.32 | | | | | | | | (0.23 | | | | | – | | | | 0.42) | | | | | |  | | | | | 0.80 | | | | | | (0.73 | | | | | – | | 0.87) | | | | |  | | | | | | 0.59 | | | | | (0.52 | | | | – | | | | 0.65) | | |  | | | | | | 0.57 | | | | (0.43 | | | | | | – | | 0.69) | | | | | | |  | | | 0.60 | | | | | (0.52 | | | | | – | | | | | | | 0.67) | | | |  | | | | 1.13 | | |  | |
|  | | **Speed OR Upper gutter surface** | | | | | | 239 | | | | |  | | | | 92 | | | 96 | | | | 14 | | | 37 | | | |  | | | | | 0.87 | | | | | | | | (0.79 | | | | | – | | | | 0.93) | | | | | |  | | | | | 0.28 | | | | | | (0.20 | | | | | – | | 0.36) | | | | |  | | | | | | 0.54 | | | | | (0.47 | | | | – | | | | 0.60) | | |  | | | | | | 0.49 | | | | (0.42 | | | | | | – | | 0.56) | | | | | | |  | | | 0.73 | | | | | (0.58 | | | | | – | | | | | | | 0.84) | | | |  | | | | 1.15 | | |  | |
|  | | **Speed AND Gutter surface** | | | | | | 239 | | | | |  | | | | 81 | | | 88 | | | | 25 | | | 45 | | | |  | | | | | 0.76 | | | | | | | | (0.67 | | | | | – | | | | 0.84) | | | | | |  | | | | | 0.34 | | | | | | (0.26 | | | | | – | | 0.43) | | | | |  | | | | | | 0.53 | | | | | (0.46 | | | | – | | | | 0.59) | | |  | | | | | | 0.48 | | | | (0.40 | | | | | | – | | 0.56) | | | | | | |  | | | 0.64 | | | | | (0.52 | | | | | – | | | | | | | 0.75) | | | |  | | | | 1.10 | | |  | |
|  | | **Speed OR Gutter surface** | | | | | | 239 | | | | |  | | | | 98 | | | 105 | | | | 8 | | | 28 | | | |  | | | | | 0.92 | | | | | | | | (0.86 | | | | | – | | | | 0.97) | | | | | |  | | | | | 0.21 | | | | | | (0.14 | | | | | – | | 0.29) | | | | |  | | | | | | 0.53 | | | | | (0.46 | | | | – | | | | 0.59) | | |  | | | | | | 0.48 | | | | (0.41 | | | | | | – | | 0.55) | | | | | | |  | | | 0.78 | | | | | (0.61 | | | | | – | | | | | | | 0.90) | | | |  | | | | 1.14 | | |  | |
|  | |  | | | | | |  | | | | |  | | | |  | | |  | | | |  | | |  | | | |  | | | | |  | | | | | | | |  | | | | |  | | | |  | | | | | |  | | | | |  | | | | | |  | | | | |  | |  | | | | |  | | | | | |  | | | | |  | | | |  | | | |  | | |  | | | | | |  | | | |  | | | | | |  | |  | | | | | | |  | | |  | | | | |  | | | | |  | | | | | | |  | | | |  | | | |  | | |  | |
|  | | **Yergason AND Hypervascularisation** | | | | | | 242 | | | | |  | | | | 2 | | | 3 | | | | 106 | | | 131 | | | |  | | | | | 0.02 | | | | | | | | (0.00 | | | | | – | | | | 0.07) | | | | | |  | | | | | 0.98 | | | | | | (0.94 | | | | | – | | 1.00) | | | | |  | | | | | | 0.55 | | | | | (0.48 | | | | – | | | | 0.61) | | |  | | | | | | 0.40 | | | | (0.05 | | | | | | – | | 0.85) | | | | | | |  | | | 0.55 | | | | | (0.49 | | | | | – | | | | | | | 0.62) | | | |  | | | | 1.00 | | |  | |
|  | | **Yergason OR Hypervascularisation** | | | | | | 242 | | | | |  | | | | 41 | | | 46 | | | | 67 | | | 88 | | | |  | | | | | 0.38 | | | | | | | | (0.29 | | | | | – | | | | 0.48) | | | | | |  | | | | | 0.66 | | | | | | (0.57 | | | | | – | | 0.74) | | | | |  | | | | | | 0.53 | | | | | (0.47 | | | | – | | | | 0.60) | | |  | | | | | | 0.47 | | | | (0.36 | | | | | | – | | 0.58) | | | | | | |  | | | 0.57 | | | | | (0.49 | | | | | – | | | | | | | 0.65) | | | |  | | | | 1.04 | | |  | |
|  | | **Yergason AND Upper gutter signal** | | | | | | 240 | | | | |  | | | | 17 | | | 20 | | | | 90 | | | 113 | | | |  | | | | | 0.16 | | | | | | | | (0.10 | | | | | – | | | | 0.24) | | | | | |  | | | | | 0.85 | | | | | | (0.78 | | | | | – | | 0.91) | | | | |  | | | | | | 0.54 | | | | | (0.48 | | | | – | | | | 0.61) | | |  | | | | | | 0.46 | | | | (0.29 | | | | | | – | | 0.63) | | | | | | |  | | | 0.56 | | | | | (0.49 | | | | | – | | | | | | | 0.63) | | | |  | | | | 1.01 | | |  | |
|  | | **Yergason OR Upper gutter signal** | | | | | | 240 | | | | |  | | | | 72 | | | 82 | | | | 35 | | | 51 | | | |  | | | | | 0.67 | | | | | | | | (0.58 | | | | | – | | | | 0.76) | | | | | |  | | | | | 0.38 | | | | | | (0.30 | | | | | – | | 0.47) | | | | |  | | | | | | 0.51 | | | | | (0.45 | | | | – | | | | 0.58) | | |  | | | | | | 0.47 | | | | (0.39 | | | | | | – | | 0.55) | | | | | | |  | | | 0.59 | | | | | (0.48 | | | | | – | | | | | | | 0.70) | | | |  | | | | 1.06 | | |  | |
|  | | **Yergason AND Gutter signal** | | | | | | 243 | | | | |  | | | | 15 | | | 21 | | | | 93 | | | 114 | | | |  | | | | | 0.14 | | | | | | | | (0.08 | | | | | – | | | | 0.22) | | | | | |  | | | | | 0.84 | | | | | | (0.77 | | | | | – | | 0.90) | | | | |  | | | | | | 0.53 | | | | | (0.47 | | | | – | | | | 0.59) | | |  | | | | | | 0.42 | | | | (0.26 | | | | | | – | | 0.59) | | | | | | |  | | | 0.55 | | | | | (0.48 | | | | | – | | | | | | | 0.62) | | | |  | | | | 0.98 | | |  | |
|  | | **Yergason OR Gutter signal** | | | | | | 243 | | | | |  | | | | 68 | | | 78 | | | | 40 | | | 57 | | | |  | | | | | 0.63 | | | | | | | | (0.53 | | | | | – | | | | 0.72) | | | | | |  | | | | | 0.42 | | | | | | (0.34 | | | | | – | | 0.51) | | | | |  | | | | | | 0.51 | | | | | (0.45 | | | | – | | | | 0.58) | | |  | | | | | | 0.47 | | | | (0.38 | | | | | | – | | 0.55) | | | | | | |  | | | 0.59 | | | | | (0.48 | | | | | – | | | | | | | 0.69) | | | |  | | | | 1.05 | | |  | |
|  | | **Yergason AND Upper gutter position** | | | | | | 241 | | | | |  | | | | 35 | | | 39 | | | | 71 | | | 96 | | | |  | | | | | 0.33 | | | | | | | | (0.24 | | | | | – | | | | 0.43) | | | | | |  | | | | | 0.71 | | | | | | (0.63 | | | | | – | | 0.79) | | | | |  | | | | | | 0.54 | | | | | (0.48 | | | | – | | | | 0.61) | | |  | | | | | | 0.47 | | | | (0.36 | | | | | | – | | 0.59) | | | | | | |  | | | 0.57 | | | | | (0.50 | | | | | – | | | | | | | 0.65) | | | |  | | | | 1.04 | | |  | |
|  | | **Yergason OR Upper gutter position** | | | | | | 241 | | | | |  | | | | 106 | | | 135 | | | | 0 | | | 0 | | | |  | | | | | 1.00 | | | | | | | | (0.97 | | | | | – | | | | 1.00) | | | | | |  | | | | | 0.00 | | | | | | (0.00 | | | | | – | | 0.03) | | | | |  | | | | | | 0.44 | | | | | (0.38 | | | | – | | | | 0.50) | | |  | | | | | | 0.44 | | | | (0.38 | | | | | | – | | 0.50) | | | | | | |  | | | NaN | | | | | (0.00 | | | | | – | | | | | | | 1.00) | | | |  | | | | 1.00 | | |  | |
|  | | **Yergason AND Gutter position** | | | | | | 242 | | | | |  | | | | 3 | | | 0 | | | | 104 | | | 135 | | | |  | | | | | 0.03 | | | | | | | | (0.01 | | | | | – | | | | 0.08) | | | | | |  | | | | | 1.00 | | | | | | (0.97 | | | | | – | | 1.00) | | | | |  | | | | | | 0.57 | | | | | (0.51 | | | | – | | | | 0.63) | | |  | | | | | | 1.00 | | | | (0.29 | | | | | | – | | 1.00) | | | | | | |  | | | 0.56 | | | | | (0.50 | | | | | – | | | | | | | 0.63) | | | |  | | | | 1.03 | | |  | |
|  | | **Yergason OR Gutter position** | | | | | | 242 | | | | |  | | | | 46 | | | 45 | | | | 61 | | | 90 | | | |  | | | | | 0.43 | | | | | | | | (0.33 | | | | | – | | | | 0.53) | | | | | |  | | | | | 0.67 | | | | | | (0.58 | | | | | – | | 0.75) | | | | |  | | | | | | 0.56 | | | | | (0.50 | | | | – | | | | 0.63) | | |  | | | | | | 0.51 | | | | (0.40 | | | | | | – | | 0.61) | | | | | | |  | | | 0.60 | | | | | (0.51 | | | | | – | | | | | | | 0.68) | | | |  | | | | 1.10 | | |  | |
|  | | **Yergason AND Upper gutter surface** | | | | | | 237 | | | | |  | | | | 13 | | | 10 | | | | 92 | | | 122 | | | |  | | | | | 0.12 | | | | | | | | (0.07 | | | | | – | | | | 0.20) | | | | | |  | | | | | 0.92 | | | | | | (0.87 | | | | | – | | 0.96) | | | | |  | | | | | | 0.57 | | | | | (0.50 | | | | – | | | | 0.63) | | |  | | | | | | 0.57 | | | | (0.34 | | | | | | – | | 0.77) | | | | | | |  | | | 0.57 | | | | | (0.50 | | | | | – | | | | | | | 0.64) | | | |  | | | | 1.05 | | |  | |
|  | | **Yergason OR Upper gutter surface** | | | | | | 237 | | | | |  | | | | 66 | | | 62 | | | | 39 | | | 70 | | | |  | | | | | 0.63 | | | | | | | | (0.53 | | | | | – | | | | 0.72) | | | | | |  | | | | | 0.53 | | | | | | (0.44 | | | | | – | | 0.62) | | | | |  | | | | | | 0.57 | | | | | (0.51 | | | | – | | | | 0.64) | | |  | | | | | | 0.52 | | | | (0.43 | | | | | | – | | 0.60) | | | | | | |  | | | 0.64 | | | | | (0.54 | | | | | – | | | | | | | 0.73) | | | |  | | | | 1.16 | | |  | |
|  | | **Yergason AND Gutter surface** | | | | | | 237 | | | | |  | | | | 35 | | | 39 | | | | 70 | | | 93 | | | |  | | | | | 0.33 | | | | | | | | (0.24 | | | | | – | | | | 0.43) | | | | | |  | | | | | 0.70 | | | | | | (0.62 | | | | | – | | 0.78) | | | | |  | | | | | | 0.54 | | | | | (0.47 | | | | – | | | | 0.60) | | |  | | | | | | 0.47 | | | | (0.36 | | | | | | – | | 0.59) | | | | | | |  | | | 0.57 | | | | | (0.49 | | | | | – | | | | | | | 0.65) | | | |  | | | | 1.04 | | |  | |
|  | | **Yergason OR Gutter surface** | | | | | | 237 | | | | |  | | | | 80 | | | 83 | | | | 25 | | | 49 | | | |  | | | | | 0.76 | | | | | | | | (0.67 | | | | | – | | | | 0.84) | | | | | |  | | | | | 0.37 | | | | | | (0.29 | | | | | – | | 0.46) | | | | |  | | | | | | 0.54 | | | | | (0.48 | | | | – | | | | 0.61) | | |  | | | | | | 0.49 | | | | (0.41 | | | | | | – | | 0.57) | | | | | | |  | | | 0.66 | | | | | (0.54 | | | | | – | | | | | | | 0.77) | | | |  | | | | 1.13 | | |  | |
|  | |  | | | | | |  | | | | |  | | | |  | | |  | | | |  | | |  | | | |  | | | | |  | | | | | | | |  | | | | |  | | | |  | | | | | |  | | | | |  | | | | | |  | | | | |  | |  | | | | |  | | | | | |  | | | | |  | | | |  | | | |  | | |  | | | | | |  | | | |  | | | | | |  | |  | | | | | | |  | | |  | | | | |  | | | | |  | | | | | | |  | | | |  | | | |  | | |  | |
|  | | **Kibler AND Hypervascularisation** | | | | | | 242 | | | | |  | | | | 3 | | | 4 | | | | 104 | | | 131 | | | |  | | | | | 0.03 | | | | | | | | (0.01 | | | | | – | | | | 0.08) | | | | | |  | | | | | 0.97 | | | | | | (0.93 | | | | | – | | 0.99) | | | | |  | | | | | | 0.55 | | | | | (0.49 | | | | – | | | | 0.62) | | |  | | | | | | 0.43 | | | | (0.10 | | | | | | – | | 0.82) | | | | | | |  | | | 0.56 | | | | | (0.49 | | | | | – | | | | | | | 0.62) | | | |  | | | | 1.00 | | |  | |
|  | | **Kibler OR Hypervascularisation** | | | | | | 242 | | | | |  | | | | 64 | | | 57 | | | | 43 | | | 78 | | | |  | | | | | 0.60 | | | | | | | | (0.50 | | | | | – | | | | 0.69) | | | | | |  | | | | | 0.58 | | | | | | (0.49 | | | | | – | | 0.66) | | | | |  | | | | | | 0.59 | | | | | (0.52 | | | | – | | | | 0.65) | | |  | | | | | | 0.53 | | | | (0.44 | | | | | | – | | 0.62) | | | | | | |  | | | 0.64 | | | | | (0.55 | | | | | – | | | | | | | 0.73) | | | |  | | | | 1.18 | | |  | |
|  | | **Kibler AND Upper gutter signal** | | | | | | 240 | | | | |  | | | | 34 | | | 23 | | | | 72 | | | 111 | | | |  | | | | | 0.32 | | | | | | | | (0.23 | | | | | – | | | | 0.42) | | | | | |  | | | | | 0.83 | | | | | | (0.75 | | | | | – | | 0.89) | | | | |  | | | | | | 0.60 | | | | | (0.54 | | | | – | | | | 0.67) | | |  | | | | | | 0.60 | | | | (0.46 | | | | | | – | | 0.72) | | | | | | |  | | | 0.61 | | | | | (0.53 | | | | | – | | | | | | | 0.68) | | | |  | | | | 1.15 | | |  | |
|  | | **Kibler OR Upper gutter signal** | | | | | | 240 | | | | |  | | | | 78 | | | 91 | | | | 28 | | | 43 | | | |  | | | | | 0.74 | | | | | | | | (0.64 | | | | | – | | | | 0.82) | | | | | |  | | | | | 0.32 | | | | | | (0.24 | | | | | – | | 0.41) | | | | |  | | | | | | 0.50 | | | | | (0.44 | | | | – | | | | 0.57) | | |  | | | | | | 0.46 | | | | (0.38 | | | | | | – | | 0.54) | | | | | | |  | | | 0.61 | | | | | (0.48 | | | | | – | | | | | | | 0.72) | | | |  | | | | 1.06 | | |  | |
|  | | **Kibler AND Gutter signal** | | | | | | 243 | | | | |  | | | | 29 | | | 21 | | | | 78 | | | 115 | | | |  | | | | | 0.27 | | | | | | | | (0.19 | | | | | – | | | | 0.37) | | | | | |  | | | | | 0.85 | | | | | | (0.77 | | | | | – | | 0.90) | | | | |  | | | | | | 0.59 | | | | | (0.53 | | | | – | | | | 0.65) | | |  | | | | | | 0.58 | | | | (0.43 | | | | | | – | | 0.72) | | | | | | |  | | | 0.60 | | | | | (0.52 | | | | | – | | | | | | | 0.67) | | | |  | | | | 1.12 | | |  | |
|  | | **Kibler OR Gutter signal** | | | | | | 243 | | | | |  | | | | 78 | | | 90 | | | | 29 | | | 46 | | | |  | | | | | 0.73 | | | | | | | | (0.63 | | | | | – | | | | 0.81) | | | | | |  | | | | | 0.34 | | | | | | (0.26 | | | | | – | | 0.42) | | | | |  | | | | | | 0.51 | | | | | (0.45 | | | | – | | | | 0.57) | | |  | | | | | | 0.46 | | | | (0.39 | | | | | | – | | 0.54) | | | | | | |  | | | 0.61 | | | | | (0.49 | | | | | – | | | | | | | 0.72) | | | |  | | | | 1.07 | | |  | |
|  | | **Kibler AND Upper gutter position** | | | | | | 241 | | | | |  | | | | 58 | | | 51 | | | | 47 | | | 85 | | | |  | | | | | 0.55 | | | | | | | | (0.45 | | | | | – | | | | 0.65) | | | | | |  | | | | | 0.63 | | | | | | (0.54 | | | | | – | | 0.71) | | | | |  | | | | | | 0.59 | | | | | (0.53 | | | | – | | | | 0.66) | | |  | | | | | | 0.53 | | | | (0.43 | | | | | | – | | 0.63) | | | | | | |  | | | 0.64 | | | | | (0.56 | | | | | – | | | | | | | 0.73) | | | |  | | | | 1.18 | | |  | |
|  | | **Kibler OR Upper gutter position** | | | | | | 241 | | | | |  | | | | 105 | | | 136 | | | | 0 | | | 0 | | | |  | | | | | 1.00 | | | | | | | | (0.97 | | | | | – | | | | 1.00) | | | | | |  | | | | | 0.00 | | | | | | (0.00 | | | | | – | | 0.03) | | | | |  | | | | | | 0.44 | | | | | (0.37 | | | | – | | | | 0.50) | | |  | | | | | | 0.44 | | | | (0.37 | | | | | | – | | 0.50) | | | | | | |  | | | NaN | | | | | (0.00 | | | | | – | | | | | | | 1.00) | | | |  | | | | 1.00 | | |  | |
|  | | **Kibler AND Gutter position** | | | | | | 242 | | | | |  | | | | 8 | | | 1 | | | | 98 | | | 135 | | | |  | | | | | 0.08 | | | | | | | | (0.03 | | | | | – | | | | 0.14) | | | | | |  | | | | | 0.99 | | | | | | (0.96 | | | | | – | | 1.00) | | | | |  | | | | | | 0.59 | | | | | (0.53 | | | | – | | | | 0.65) | | |  | | | | | | 0.89 | | | | (0.52 | | | | | | – | | 1.00) | | | | | | |  | | | 0.58 | | | | | (0.51 | | | | | – | | | | | | | 0.64) | | | |  | | | | 1.07 | | |  | |
|  | | **Kibler OR Gutter position** | | | | | | 242 | | | | |  | | | | 65 | | | 56 | | | | 41 | | | 80 | | | |  | | | | | 0.61 | | | | | | | | (0.51 | | | | | – | | | | 0.71) | | | | | |  | | | | | 0.59 | | | | | | (0.50 | | | | | – | | 0.67) | | | | |  | | | | | | 0.60 | | | | | (0.53 | | | | – | | | | 0.66) | | |  | | | | | | 0.54 | | | | (0.44 | | | | | | – | | 0.63) | | | | | | |  | | | 0.66 | | | | | (0.57 | | | | | – | | | | | | | 0.74) | | | |  | | | | 1.20 | | |  | |
|  | | **Kibler AND Upper gutter surface** | | | | | | 237 | | | | |  | | | | 22 | | | 15 | | | | 82 | | | 118 | | | |  | | | | | 0.21 | | | | | | | | (0.14 | | | | | – | | | | 0.30) | | | | | |  | | | | | 0.89 | | | | | | (0.82 | | | | | – | | 0.94) | | | | |  | | | | | | 0.59 | | | | | (0.53 | | | | – | | | | 0.65) | | |  | | | | | | 0.59 | | | | (0.42 | | | | | | – | | 0.75) | | | | | | |  | | | 0.59 | | | | | (0.52 | | | | | – | | | | | | | 0.66) | | | |  | | | | 1.10 | | |  | |
|  | | **Kibler OR Upper gutter surface** | | | | | | 237 | | | | |  | | | | 80 | | | 69 | | | | 24 | | | 64 | | | |  | | | | | 0.77 | | | | | | | | (0.68 | | | | | – | | | | 0.85) | | | | | |  | | | | | 0.48 | | | | | | (0.39 | | | | | – | | 0.57) | | | | |  | | | | | | 0.61 | | | | | (0.54 | | | | – | | | | 0.67) | | |  | | | | | | 0.54 | | | | (0.45 | | | | | | – | | 0.62) | | | | | | |  | | | 0.73 | | | | | (0.62 | | | | | – | | | | | | | 0.82) | | | |  | | | | 1.25 | | |  | |
|  | | **Kibler AND Gutter surface** | | | | | | 237 | | | | |  | | | | 58 | | | 50 | | | | 46 | | | 83 | | | |  | | | | | 0.56 | | | | | | | | (0.46 | | | | | – | | | | 0.66) | | | | | |  | | | | | 0.62 | | | | | | (0.54 | | | | | – | | 0.71) | | | | |  | | | | | | 0.59 | | | | | (0.53 | | | | – | | | | 0.66) | | |  | | | | | | 0.54 | | | | (0.44 | | | | | | – | | 0.63) | | | | | | |  | | | 0.64 | | | | | (0.55 | | | | | – | | | | | | | 0.73) | | | |  | | | | 1.18 | | |  | |
|  | | **Kibler OR Gutter surface** | | | | | | 237 | | | | |  | | | | 88 | | | 86 | | | | 16 | | | 47 | | | |  | | | | | 0.85 | | | | | | | | (0.76 | | | | | – | | | | 0.91) | | | | | |  | | | | | 0.35 | | | | | | (0.27 | | | | | – | | 0.44) | | | | |  | | | | | | 0.57 | | | | | (0.50 | | | | – | | | | 0.63) | | |  | | | | | | 0.51 | | | | (0.43 | | | | | | – | | 0.58) | | | | | | |  | | | 0.75 | | | | | (0.62 | | | | | – | | | | | | | 0.85) | | | |  | | | | 1.20 | | |  | |
|  | |  | | | | | |  | | | | |  | | | |  | | |  | | | |  | | |  | | | |  | | | | |  | | | | | | | |  | | | | |  | | | |  | | | | | |  | | | | |  | | | | | |  | | | | |  | |  | | | | |  | | | | | |  | | | | |  | | | |  | | | |  | | |  | | | | | |  | | | |  | | | | | |  | |  | | | | | | |  | | |  | | | | |  | | | | |  | | | | | | |  | | | |  | | | |  | | |  | |
|  | | **Tenderness AND Hypervascularisation** | | | | | | 240 | | | | |  | | | | 5 | | | 8 | | | | 101 | | | 126 | | | |  | | | | | 0.05 | | | | | | | | (0.02 | | | | | – | | | | 0.11) | | | | | |  | | | | | 0.94 | | | | | | (0.89 | | | | | – | | 0.97) | | | | |  | | | | | | 0.55 | | | | | (0.48 | | | | – | | | | 0.61) | | |  | | | | | | 0.38 | | | | (0.14 | | | | | | – | | 0.68) | | | | | | |  | | | 0.56 | | | | | (0.49 | | | | | – | | | | | | | 0.62) | | | |  | | | | 0.99 | | |  | |
|  | | **Tenderness OR Hypervascularisation** | | | | | | 240 | | | | |  | | | | 82 | | | 80 | | | | 24 | | | 54 | | | |  | | | | | 0.77 | | | | | | | | (0.68 | | | | | – | | | | 0.85) | | | | | |  | | | | | 0.40 | | | | | | (0.32 | | | | | – | | 0.49) | | | | |  | | | | | | 0.57 | | | | | (0.50 | | | | – | | | | 0.63) | | |  | | | | | | 0.51 | | | | (0.43 | | | | | | – | | 0.59) | | | | | | |  | | | 0.69 | | | | | (0.58 | | | | | – | | | | | | | 0.79) | | | |  | | | | 1.18 | | |  | |
|  | | **Tenderness AND Upper gutter signal** | | | | | | 238 | | | | |  | | | | 41 | | | 38 | | | | 64 | | | 95 | | | |  | | | | | 0.39 | | | | | | | | (0.30 | | | | | – | | | | 0.49) | | | | | |  | | | | | 0.71 | | | | | | (0.63 | | | | | – | | 0.79) | | | | |  | | | | | | 0.57 | | | | | (0.51 | | | | – | | | | 0.64) | | |  | | | | | | 0.52 | | | | (0.40 | | | | | | – | | 0.63) | | | | | | |  | | | 0.60 | | | | | (0.52 | | | | | – | | | | | | | 0.67) | | | |  | | | | 1.10 | | |  | |
|  | | **Tenderness OR Upper gutter signal** | | | | | | 238 | | | | |  | | | | 91 | | | 103 | | | | 14 | | | 30 | | | |  | | | | | 0.87 | | | | | | | | (0.79 | | | | | – | | | | 0.93) | | | | | |  | | | | | 0.23 | | | | | | (0.16 | | | | | – | | 0.31) | | | | |  | | | | | | 0.51 | | | | | (0.44 | | | | – | | | | 0.57) | | |  | | | | | | 0.47 | | | | (0.40 | | | | | | – | | 0.54) | | | | | | |  | | | 0.68 | | | | | (0.52 | | | | | – | | | | | | | 0.81) | | | |  | | | | 1.09 | | |  | |
|  | | **Tenderness AND Gutter signal** | | | | | | 241 | | | | |  | | | | 35 | | | 36 | | | | 71 | | | 99 | | | |  | | | | | 0.33 | | | | | | | | (0.24 | | | | | – | | | | 0.43) | | | | | |  | | | | | 0.73 | | | | | | (0.65 | | | | | – | | 0.81) | | | | |  | | | | | | 0.56 | | | | | (0.49 | | | | – | | | | 0.62) | | |  | | | | | | 0.49 | | | | (0.37 | | | | | | – | | 0.61) | | | | | | |  | | | 0.58 | | | | | (0.50 | | | | | – | | | | | | | 0.66) | | | |  | | | | 1.06 | | |  | |
|  | | **Tenderness OR Gutter signal** | | | | | | 241 | | | | |  | | | | 92 | | | 102 | | | | 14 | | | 33 | | | |  | | | | | 0.87 | | | | | | | | (0.79 | | | | | – | | | | 0.93) | | | | | |  | | | | | 0.24 | | | | | | (0.17 | | | | | – | | 0.33) | | | | |  | | | | | | 0.52 | | | | | (0.45 | | | | – | | | | 0.58) | | |  | | | | | | 0.47 | | | | (0.40 | | | | | | – | | 0.55) | | | | | | |  | | | 0.70 | | | | | (0.55 | | | | | – | | | | | | | 0.83) | | | |  | | | | 1.11 | | |  | |
|  | | **Tenderness AND Upper gutter position** | | | | | | 239 | | | | |  | | | | 78 | | | 78 | | | | 26 | | | 57 | | | |  | | | | | 0.75 | | | | | | | | (0.66 | | | | | – | | | | 0.83) | | | | | |  | | | | | 0.42 | | | | | | (0.34 | | | | | – | | 0.51) | | | | |  | | | | | | 0.56 | | | | | (0.50 | | | | – | | | | 0.63) | | |  | | | | | | 0.50 | | | | (0.42 | | | | | | – | | 0.58) | | | | | | |  | | | 0.69 | | | | | (0.58 | | | | | – | | | | | | | 0.78) | | | |  | | | | 1.17 | | |  | |
|  | | **Tenderness OR Upper gutter position** | | | | | | 239 | | | | |  | | | | 104 | | | 135 | | | | 0 | | | 0 | | | |  | | | | | 1.00 | | | | | | | | (0.97 | | | | | – | | | | 1.00) | | | | | |  | | | | | 0.00 | | | | | | (0.00 | | | | | – | | 0.03) | | | | |  | | | | | | 0.44 | | | | | (0.37 | | | | – | | | | 0.50) | | |  | | | | | | 0.44 | | | | (0.44 | | | | | | – | | 0.50) | | | | | | |  | | | NaN | | | | | (0.00 | | | | | – | | | | | | | 1.00) | | | |  | | | | 1.00 | | |  | |
|  | | **Tenderness AND Gutter position** | | | | | | 240 | | | | |  | | | | 12 | | | 3 | | | | 93 | | | 132 | | | |  | | | | | 0.11 | | | | | | | | (0.06 | | | | | – | | | | 0.19) | | | | | |  | | | | | 0.98 | | | | | | (0.94 | | | | | – | | 1.00) | | | | |  | | | | | | 0.60 | | | | | (0.54 | | | | – | | | | 0.66) | | |  | | | | | | 0.80 | | | | (0.52 | | | | | | – | | 0.96) | | | | | | |  | | | 0.59 | | | | | (0.52 | | | | | – | | | | | | | 0.65) | | | |  | | | | 1.09 | | |  | |
|  | | **Tenderness OR Gutter position** | | | | | | 240 | | | | |  | | | | 81 | | | 81 | | | | 24 | | | 54 | | | |  | | | | | 0.77 | | | | | | | | (0.68 | | | | | – | | | | 0.85) | | | | | |  | | | | | 0.40 | | | | | | (0.32 | | | | | – | | 0.49) | | | | |  | | | | | | 0.56 | | | | | (0.50 | | | | – | | | | 0.63) | | |  | | | | | | 0.50 | | | | (0.42 | | | | | | – | | 0.58) | | | | | | |  | | | 0.69 | | | | | (0.58 | | | | | – | | | | | | | 0.79) | | | |  | | | | 1.17 | | |  | |
|  | | **Tenderness AND Upper gutter surface** | | | | | | 235 | | | | |  | | | | 31 | | | 23 | | | | 72 | | | 109 | | | |  | | | | | 0.30 | | | | | | | | (0.21 | | | | | – | | | | 0.40) | | | | | |  | | | | | 0.83 | | | | | | (0.75 | | | | | – | | 0.89) | | | | |  | | | | | | 0.60 | | | | | (0.53 | | | | – | | | | 0.66) | | |  | | | | | | 0.57 | | | | (0.43 | | | | | | – | | 0.71) | | | | | | |  | | | 0.60 | | | | | (0.53 | | | | | – | | | | | | | 0.67) | | | |  | | | | 1.13 | | |  | |
|  | | **Tenderness OR Upper gutter surface** | | | | | | 235 | | | | |  | | | | 90 | | | 88 | | | | 13 | | | 44 | | | |  | | | | | 0.87 | | | | | | | | (0.79 | | | | | – | | | | 0.93) | | | | | |  | | | | | 0.33 | | | | | | (0.25 | | | | | – | | 0.42) | | | | |  | | | | | | 0.57 | | | | | (0.50 | | | | – | | | | 0.63) | | |  | | | | | | 0.51 | | | | (0.43 | | | | | | – | | 0.58) | | | | | | |  | | | 0.77 | | | | | (0.64 | | | | | – | | | | | | | 0.87) | | | |  | | | | 1.21 | | |  | |
|  | | **Tenderness AND Gutter surface** | | | | | | 235 | | | | |  | | | | 77 | | | 77 | | | | 26 | | | 55 | | | |  | | | | | 0.75 | | | | | | | | (0.65 | | | | | – | | | | 0.83) | | | | | |  | | | | | 0.42 | | | | | | (0.33 | | | | | – | | 0.51) | | | | |  | | | | | | 0.56 | | | | | (0.50 | | | | – | | | | 0.63) | | |  | | | | | | 0.50 | | | | (0.42 | | | | | | – | | 0.58) | | | | | | |  | | | 0.68 | | | | | (0.57 | | | | | – | | | | | | | 0.78) | | | |  | | | | 1.16 | | |  | |
|  | | **Tenderness OR Gutter surface** | | | | | | 235 | | | | |  | | | | 96 | | | 99 | | | | 7 | | | 33 | | | |  | | | | | 0.93 | | | | | | | | (0.86 | | | | | – | | | | 0.97) | | | | | |  | | | | | 0.25 | | | | | | (0.18 | | | | | – | | 0.33) | | | | |  | | | | | | 0.55 | | | | | (0.48 | | | | – | | | | 0.61) | | |  | | | | | | 0.49 | | | | (0.42 | | | | | | – | | 0.56) | | | | | | |  | | | 0.83 | | | | | (0.67 | | | | | – | | | | | | | 0.93) | | | |  | | | | 1.18 | | |  | |
|  | | | | |  |  | | | |  | | | |  | | | |  | | | | |  | | | | |  | | | | | | | | | |  | | | |  | | | | | |  | | | | | | |  | | |  | | | | | | | | | | |  | | | | |  | | | | |  | | | | | | | |  | | | |  | | | | |  | | | | | |  | | | | | | |  | | | | | |  | | |  | | | | | | |  | | | | | |  | | | | |  | | | | | | |  | | | | | | |  | | | | | |  | | |  | | |
| *Abbreviations: Est., Estimation; FN, False Negative; FP, False Positive; N, Cohort size; NPV, Negative Predictive Value; PPV, Positive Predictive Value; Se, Sensitivity; Sp, Specificity; TN, True Negative; TP, True Positive;* | | | | | | | | | | | | | | | | | | | | | | | | | | | | | | | | | | | | | | | | | | | | | | | | | | | | | | | | | | | | | | | | | | | | | | | | | | | | | | | | | | | | | | | | | | | | | | | | | | | | | | | | | | | | | | | | | | | | | | | | | | | | | | | | | | | | | | | | | | | | | | | | | | | | | | | | | | | | | | | | | |
